# A niche-mimicking polymer hydrogel-based approach to identify molecular targets for tackling human pancreatic cancer stem cells

**DOI:** 10.1186/s41232-023-00296-0

**Published:** 2023-09-27

**Authors:** Yoshitaka Murota, Mariko Nagane, Mei Wu, Mithun Santra, Seshasailam Venkateswaran, Shinji Tanaka, Mark Bradley, Tetsuya Taga, Kouichi Tabu

**Affiliations:** 1 Department of Stem Cell Regulation, Medical Research Institute, Tokyo Medical and Dental University (TMDU), 1-5-45, Yushima, Bunkyo-Ku, Tokyo, 113-8510 Japan; 2 School of Chemistry, University of Edinburgh, Joseph Black Building, West Mains Road, Edinburgh, EH9 3FJ UK; 3 Department of Molecular Oncology, Graduate School of Medicine, Tokyo Medical and Dental University (TMDU), 1-5-45 Yushima, Bunkyo-Ku, Tokyo, 113-8519 Japan

**Keywords:** Cancer stem cells (CSCs), Niche mimicry, Synthetic polymer, Polymer microarray, Hydrogel, Biomaterial

## Abstract

**Background:**

Pancreatic adenocarcinoma (PAAD) is one of the most fatal human cancers, but effective therapies remain to be established. Cancer stem cells (CSCs) are highly resistant to anti-cancer drugs and a deeper understanding of their microenvironmental niche has been considered important to provide understanding and solutions to cancer eradication. However, as the CSC niche is composed of a wide variety of biological and physicochemical factors, the development of multidisciplinary tools that recapitulate their complex features is indispensable. Synthetic polymers have been studied as attractive biomaterials due to their tunable biofunctionalities, while hydrogelation technique further renders upon them a diversity of physical properties, making them an attractive tool for analysis of the CSC niche.

**Methods:**

To develop innovative materials that recapitulate the CSC niche in pancreatic cancers, we performed polymer microarray analysis to identify niche-mimicking scaffolds that preferentially supported the growth of CSCs. The niche-mimicking activity of the identified polymers was further optimized by polyethylene glycol (PEG)-based hydrogelation. To reveal the biological mechanisms behind the activity of the optimized hydrogels towards CSCs, proteins binding onto the hydrogel were analyzed by liquid chromatography with tandem mass spectrometry (LC–MS/MS), and the potential therapeutic targets were validated by looking at gene expression and patients’ outcome in the TCGA database.

**Results:**

PA531, a heteropolymer composed of 2-methoxyethyl methacrylate (MEMA) and 2-(diethylamino)ethyl methacrylate (DEAEMA) (5.5:4.5) that specifically supports the growth and maintenance of CSCs was identified by polymer microarray screening using the human PAAD cell line KLM1. The polymer PA531 was converted into five hydrogels (PA531-HG1 to HG5) and developed to give an optimized scaffold with the highest CSC niche-mimicking activities. From this polymer that recapitulated CSC binding and control, the proteins fetuin-B and angiotensinogen were identified as candidate target molecules with clinical significance due to the correlation between gene expression levels and prognosis in PAAD patients and the proteins associated with the niche-mimicking polymer.

**Conclusion:**

This study screened for biofunctional polymers suitable for recapitulation of the pancreatic CSC niche and one hydrogel with high niche-mimicking abilities was successfully fabricated. Two soluble factors with clinical significance were identified as potential candidates for biomarkers and therapeutic targets in pancreatic cancers. Such a biomaterial-based approach could be a new platform in drug discovery and therapy development against CSCs, via targeting of their niche.

**Supplementary Information:**

The online version contains supplementary material available at 10.1186/s41232-023-00296-0.

## Background

Pancreatic adenocarcinoma (PAAD), the most common histologic form of pancreatic cancer, is an intractable malignancy and the incidence is increasing worldwide [1–3]. Besides difficulties in detecting at an early stage, the malignant nature is due to the presence of cancer stem cells (CSCs) responsible for metastasis, therapy resistance, and relapse, which are now widely accepted in many cancers including PAAD [4]. The cancer microenvironment is closely associated with cancer progression [5, 6] and the specialized one surrounding CSCs is called a niche [7, 8]. In light of evidence that CSCs are maintained by this niche, exploring its key components provides important clues for developing effective strategies to eradicate cancers, as disruption of the CSC niche would theoretically impair CSC developments. CSCs communicate with their niche through various kinds of cues composed of not only biological factors, e.g., vascular forming cells and infiltrating immune cells [7] but also physicochemical factors, e.g., oxygen and pH, stiffness, viscosity, and topography [9, 10]. In pancreatic cancers, inflammatory cytokines secreted from cancer-associated fibroblasts (CAFs) and bone-marrow-derived immune cells are well-characterized niche factors due to their tumor-promoting abilities via stemness-related signaling [11]. In addition, several studies have identified cathepsins, Mac-2 binding protein, fibronectin receptor, and macrophage colony stimulation factor as being secreted from pancreatic cancer cells and abundant in their microenvironment [12, 13]. From the position of their physical properties, the pancreatic cancer stroma is strictly organized compared to normal tissues and promotes epithelial-mesenchymal transition (EMT) and drug resistance, well-known characteristics of CSCs leading to metastasis and recurrence [14]. Given that the CSC niche is biologically and physiochemically complicated, the development of a ground-breaking approach that precisely recaptures these interactive networks are essential for their deep and comprehensive understanding.

Synthetic polymers are macromolecular compounds formed by the polymerization of monomers and have attracted increasing attention in the research fields of biomaterials. Polymers can display diverse mechanical, biological and chemical properties according to the monomer types, the variety of functional groups, and the conditions of polymerization [15]. Several synthetic polymers have been developed as biocompatible materials in tissue engineering [16, 17]. For example, cell sheet technology using poly(N-isopropyl acrylamide) whose hydrophilic nature changes depending on temperature, has greatly advanced basic research and developed practical solutions in regenerative medicine [18]. In addition to their clinical applications in regenerative medicine, synthetic polymers have been utilized as mechanical probes to directly elucidate cell characteristics. Engler et al. demonstrated that the stiffness of the matrix regulates the preference of mesenchymal stem cell differentiation, i.e., soft matrices induce neurogenic, stiffer ones myogenic, and rigid ones osteogenic differentiation [19]. However, applications of polymers in the field of cancer research have mainly been restricted to their use as drug carriers and delivery systems [20]. Previously we identified a urethane-based polymer (PU10), which enriches and stabilizes, a highly tumorigenic glioma stem cell (GSC) subpopulation. It is noteworthy that PU10 [poly(tetramethyleneglycol)_2,000_ and 1,3-bis(isocyanatomethyl)cyclohexane (1:1)] enriches multiple cell-/serum-derived factors, including the iron-carrier protein transferrin, the extracellular matrix-related protein galectin-1, and the thyroid hormone carrier transthyretin. This result strongly suggested that the interfacial response of cells to the polymer surface is mediated by not only the physicochemical factors but also the adsorbed proteins. Therefore, synthetic polymers have the high potential to be an innovative tool for target discovery against the CSC niche [21].

Hydrogels are soft 3-dimensional (3D) materials in which the polymer contains large amounts of water and can be considered to have even richer physical properties than 2-dimensional (2D) polymeric surfaces [22, 23]. Thus hydrogels allow modification of many biophysical and biochemical properties, including their mechanical properties, their degradation rates, architectures in terms of the proportion of water to solid, and morphologies. As such they offer opportunities to allow the discovery of new properties that have not been identified in conventional culture systems only using water-soluble cytokines and growth factors [24, 25], and thus they have the potential for the studies on the microenvironment outside cells [26]. Here, we identified a biofunctional polymer for pancreatic CSCs, with its activity optimized by PEG-based hydrogelation. Some protein factors bound to the CSC-niche mimicking hydrogel were significantly associated with prognosis in PAAD patients, demonstrating the usefulness of a biomaterial-based approach for cancer target discovery.

## Methods

### Cell culture

The human pancreatic adenocarcinoma cell line KLM1 containing the green fluorescence ZsGreen gene fused with the degron of ornithine decarboxylase (Gdeg) was maintained in RPMI1640 (Wako, Japan) supplemented with 10% fetal bovine serum (FBS), (Gibco, USA) and 50 U/ml penicillin and 50 µg streptomycin (Gibco). The green fluorescence Gdeg accumulates inside CSCs due to their low 26S proteasome activity [27]. Pancreatic CSCs isolated were from the human PAAD cell line based on the low activity of 26S proteasome (Gdeg^High^ cells) and was demonstrated to have CSC characteristics such as high tumorigenicity, drug resistance and high metastatic potential [27, 28].

### Flow cytometric analysis and cell sorting

Gdeg^High^ (ZsGreen +) and Gdeg^Low^ (ZsGreen-) KLM1 cells were separated by FACS on an Aria II (BD Biosciences, USA) equipped with a 488 nm laser. Doublet cells were removed by forward and side scatter parameters and dead cells were removed based on 2 µg/ml propidium iodide fluorescence. The fluorescence of ZsGreen was detected through a 530/30 nm band pass filter. The gating of ZsGreen + cells was decided based on the parental KLM1 cells that did not contain Gdeg.

### Polymer microarray screening

Gdeg^High^ and Gdeg^Low^ KLM1 cells were sorted and mixed at a ratio of one to one. In total 1 × 10^6^ cells were seeded onto each microarray slide set in the 4-well square dish (Nunc, USA). Two polymer microarray slides (library of polyacrylates/acrylamides/urethanes) spotted with the same series of polymers were used in this study (Slide A and B). After incubation for 18 h, the fluorescence images of ZsGreen on both slides were obtained using a BIOREVO BZ-9000 fluorescence microscope (Keyence, Japan) equipped with a BZ filter GFP-BP (excitation 470/40, emission 535/50 nm). Slide A was kept under incubation conditions for up to 48 h. Slide B was fixed with 4% PFA, stained with 5 µg/ml Hoechst 33,258 (Molecular Probes, USA) and then the fluorescence images were obtained through a BZ filter DAPI-BP (excitation 360/40, emission 460/50 nm). The fluorescence images of ZsGreen and Hoechst 33258 in Slide A were measured at 48 h like Slide B. Mean fluorescence intensities (MFIs) of ZsGreen and Hoechst 33258 in each of the polymer spots were further quantified using the BZ-II Analyzer software (Keyence). To select the functional polymers that mimic CSC niche, the values of “CSC growth rate” and “CSC specificity” were further calculated based on the following formula:

$$\mathrm{CSC\ growth\ rate}=\frac{\mathrm{ZsGreen\ intensity }\ (48\mathrm{\ hrs},\mathrm{\ Slide\ A})}{\mathrm{ZsGreen\ intensity }\ (18\mathrm{\ hrs},\mathrm{\ Slide\ A})}$$

$$\mathrm{CSC\ specificity}=\frac{ {}^{\mathrm{ZsGreen\ intensity }\ (48\mathrm{\ hrs},\mathrm{\ Slide\ A})}\!\left/ \!{}_{\mathrm{Hoechst\ intensity }\ (48\mathrm{\ hrs},\mathrm{\ Slide\ A})}\right.}{{}^{\mathrm{ZsGreen\ intensity }\ (18\mathrm{\ hrs},\mathrm{\ Slide\ B})}\!\left/ \!{}_{\mathrm{Hoechst\ intensity }\ (18\mathrm{\ hrs},\mathrm{\ Slide\ B})}\right.}$$

### Hydrogelation of hit polymers and immobilization onto the acrylated coverslips

Hydrogelation of polymers and their immobilization onto coverslips was conducted in up-scaled conditions as described previously [29]. To functionalize the glass (SiO_2_) surface, the coverslips (18-mm diameter) were etched with 1 M sodium hydroxide (NaOH) overnight and treated with a solution containing 15.4% (v/v) 3-(trimethoxysilyl)propyl methacrylate (TMSPMA) (Sigma-Aldrich, USA), 7.7% (v/v) triethylamine (Et3N) and 76.9% (v/v) acetonitrile (Sigma-Aldrich) overnight at room temperature, and then washed with acetone. Monomers composing the hit polymers were treated with aluminum oxide (Sigma-Aldrich), mixed, and Poly(ethylene glycol) diacrylate 700 (PEGDA700) crosslinker was added to the mixture at a molar ratio of 8 to 2. The photoinitiator Irgacure1173 (2-Hydroxy-2-methylpropiophenone) was then added at a concentration of 2.5% (v/v). To synthesize hydrogels, 7 µl of the monomer-PEGDA mixture was dropped onto Jetstar Inkjet art film (FORTEX, USA) made of polyethylene terephthalate (PET), and a piece of acrylated coverslip was mounted avoiding bubble formation. The coverslips were then reacted with the monomers by exposing to 254 nm UV light (3 mW/cm^2^ for 60 min) using a UV Stratalinker 1800 (Stratagene, USA). After drying at 40 ℃, the film was peeled off the immobilized polymer hydrogel-coverslips and set onto the bottom of a 12-well plastic plate. Any remaining monomers were removed and the coverslips were sterilized with 100% and 70% ethanol before cell seeding.

### Acrylamide gel electrophoresis, silver staining, and mass spectrometry analysis of hydrogel-bound proteins

KLM1 cells were cultured on the hydrogels for 5 days. All cells were removed by incubation with 0.5% trypsin–EDTA for 15 min at 37 ℃, and the protein fragments remaining on the surface of hydrogel were diluted in 100 µl of sample buffer. Complete removal of remaining cells was confirmed by nuclear staining with Hoechst 33258. Then, the samples were separated by one-dimensional sodium dodecyl sulfate–polyacrylamide gel electrophoresis (SDS-PAGE) using a SuperSep™ Ace, 5–20% gradient gel (Wako). To visualize the proteins bound from the hydrogels, silver staining was performed using a silver staining MS kit (Wako) according to the manufacturer’s protocol. To conduct proteome analysis of lysates, DetergentOUT GBS10 (G-Bioscience, USA) was used to remove SDS from the samples. The concentration of lysates after SDS removal was determined by Nanodrop (Thermo Fisher Scientific, USA). Samples were analyzed by electrospray ionization quadrupole time-of-flight (ESI-QTOF) liquid chromatography-mass spectrometer maXis-4G-CPR (Bruker, Germany). The obtained MS/MS data were searched for the Swiss-Prot database using MASCOT software ver. 2.4.1. for protein identification.

### Bioinformatic analysis

The Xena browser (http://xena.ucsc.edu/) was used to obtain RNA sequencing data and survival data of pancreatic cancer patients in the TCGA-PAAD database (accessed on 06 July 2022) [30]. Kaplan–Meier plots were generated by EZR software provided by Yoshinobu Kanda (https://www.jichi.ac.jp/saitama-sct/SaitamaHP.files/statmedEN.html) [31]. Gene set enrichment analysis (GSEA 4.3.2, https://www.gsea-msigdb.org/gsea/index.jsp, downloaded on 2023 April 27) was used to predict the significant gene sets.

### Statistics

All comparisons between experimental groups except for bio-informatic analyses were made by Student’s *t* test. Kaplan–Meier survival curves were compared by both the log-rank test and the Wilcoxon test using the EZR statistical software.

## Results

### Identification of PA531, an acrylate polymer that specifically supports the growth of CSCs

**Fig. 1:**
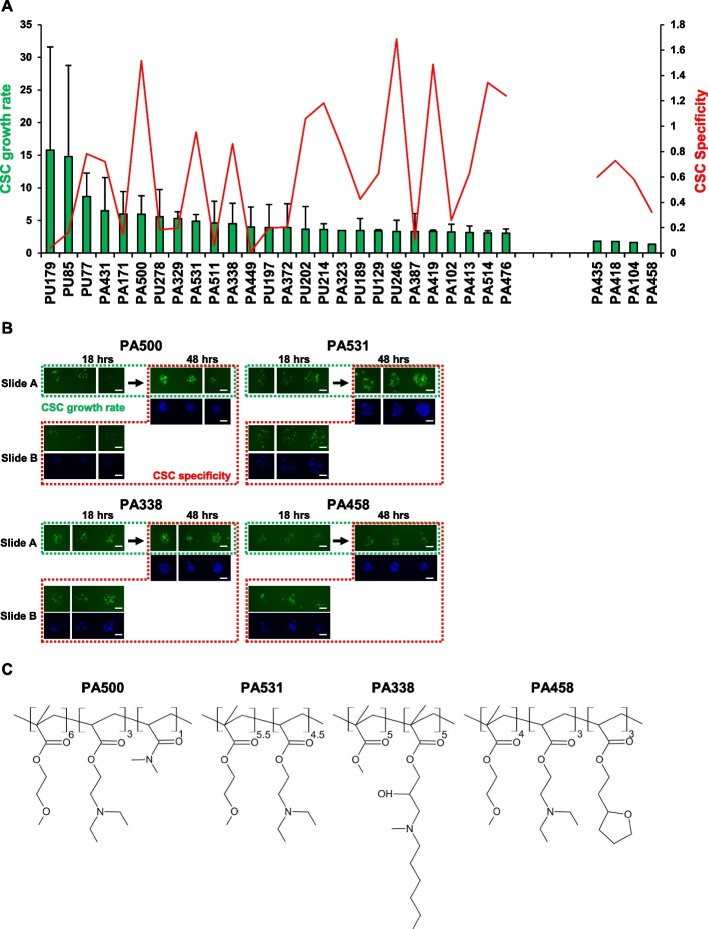
Polymer microarray analysis using pancreatic CSCs to identify niche-mimicking polymer materials. **A** Twenty-six hit polymers whose CSC growth rate was over 3 and the four control polymers. Polymer names are listed on the x-axis in the order of “CSC growth rate”, and CSC specificity values were shown in the secondary axis. All fluorescent intensity values were listed in Additional file 2. PA: Polyacrylate, PU: Polyurethane. **B** Fluorescence images of cells at 18 and 48 h after culturing on the hit and control polymers. Scale bar = 200 μm. **C** Chemical structures of the identified hit and control polymers

Comprehensive understanding of the CSC niche is limited due to its complexities and the lack of multidisciplinary methods. To overcome this problem, we firstly performed synthetic polymer microarray analysis to explore biofunctional materials for the construction of artificial CSC niche-mimicking scaffolds. Gdeg^High^ (ZsGreen +) CSCs and Gdeg^Low^ (ZsGreen-) non-CSCs were sorted from KLM1 cells and seeded onto two polymer microarray slides containing 368 polymers in triplicate (Figure S1A). When CSC growth was evaluated based on the intensities of ZsGreen at 18 and 48 h, 26 acrylate and urethane polymers (PA and PU) were identified as candidate materials with higher values of CSC growth rate than 3 (Fig. 1A). Although the mean values of the CSC growth rate were high on PU179, PU85, PU77, and PA171, fluorescence images showed a negligible number of adherent cells, so these were not polymers with increased CSCs (Figure S1B). Many cells seemed to attach to PA431 displaying relatively high values of CSC growth rate (6.48 ± 5.11) and CSC specificity (0.72), but the value of 0.72 indicates a predominant increase in non-CSCs. PA500, PA531, and PA338 were selected as hit polymers with both higher values of CSC growth rate and CSC specificity (over 1.0) as these values indicate that ZsGreen + CSCs rather than ZsGreen-non-CSCs were more predominantly proliferating on these polymers. In contrast, ZsGreen-non-CSCs preferentially proliferated on PA458, resulting in very low values of CSC growth rate (1.37 ± 0.06) and low CSC specificity (0.32) (Fig. 1A, B). PA531 is a heteropolymer composed of 2-Methoxyethyl Methacrylate (MEMA) and 2-(Diethylamino)ethyl Methacrylate (DEAEMA), PA500 of MEMA, 2-(diethylamino)ethyl acrylate (DEAEA) and N,N-dimethyl acrylamide (DMAAn), PA338 of methyl methacrylate (MMA), glycidyl methacrylate (GMA) and N,N-methylaniline (MAn), PA458 of MEMA, DEAEA and tetrahydrofurfuryl methacrylate (THFFMA) (Fig. 1C). PA500, PA531, and PA458 have similar structures containing MEMA and DEAEA, but the presence of additional monomers and a difference in their mixing ratio results in varieties of polymer functions for CSC growth and maintenance, emphasizing that polymer microarray analysis is an advanced system to screen bio-functional materials that mimic the CSC niche. Finally, we selected PA531 as the best CSC-niche mimicking polymer because the MFI of ZsGreen (an indicator of the total number of CSCs per one polymer spot) at 48 h was the highest among the three hits (Figure S1C).

### A PA531-based PEGylated hydrogel has the prominent abilities that maintain CSCs

**Fig. 2:**
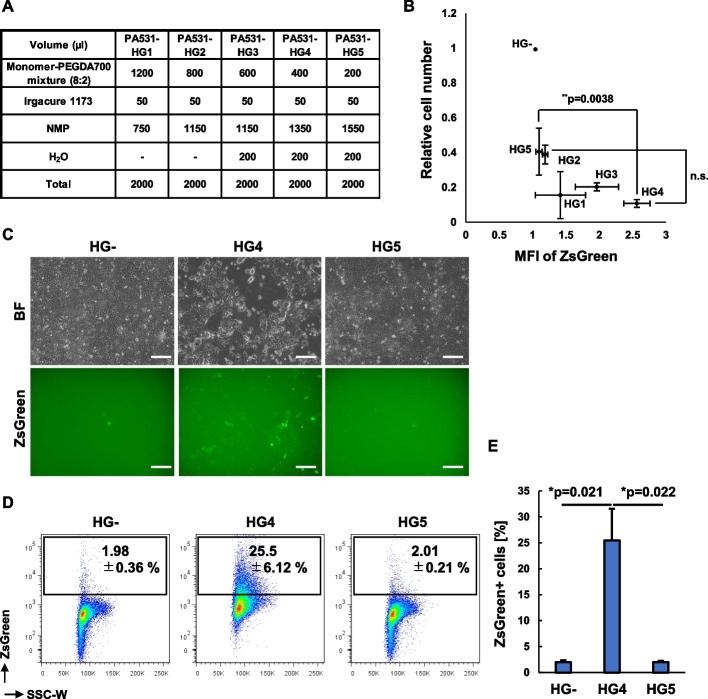
Optimization of the niche-mimicking functions of hit polymer PA531 by PEG-based hydrogelation. **A** The composition of monomers, cross-linkers, photoinitiators, NMP and water for the fabrication of five hydrogels. **B** Quantification of the mean fluorescence intensity (MFI) of ZsGreen and total cell number of KLM1-Gdeg cells cultured for 48 h on hydrogels. Data represent the averages ± standard deviation (SD) from three independent experiments. **P < 0.01, n.s: no significance. **C** Images of KLM1-Gdeg cells cultured on coverglass without hydrogel (HG-) and hit and control gels (HG4 and HG5). Scale bar = 200 μm. **D** Representative FACS plots of KLM1-Gdeg cells cultured on HG-, HG4, and HG5 for 5 days. This lot of hydrogel is different from that used for generating **B**. **E** The mean percentages ± SD of ZsGreen + cells are shown in plots and displayed in a bar graph. *P < 0.05

To confer physicochemical properties and thereby optimize the biological functions of polymers as niche mimics, we next PEGylated/hydrogelated PA531 with five different concentrations of monomers with or without H_2_O (Fig. 2A). When cells were cultured for 5 days on the five kinds of hydrogels (PA531-HG1 to HG5) immobilized onto coverslips with the total cell numbers decreased on all hydrogel-coated coverslips compared to the non-coated ones (HG-) (Fig. 2B). However among five hydrogels, HG3 and HG4 displayed relatively higher MFIs of ZsGreen, suggesting their prominent abilities as CSC niche mimicries (Fig. 2B and Figure S2A and B). In particular, HG4 more effectively maintained CSCs with statistical significance, compared to HG5 (*p* = 0.0038) (Fig. 2B) and therefore we compared these two hydrogels in the subsequent experiments. On HG5, cells firmly spread and adhered, and then highly proliferated to reach confluency. On the other hand, on HG4, some cells with high ZsGreen fluorescence seemed to be semi-adherent and showed slow growth overall. Furthermore, highly ZsGreen-expressing CSCs were present on HG4 as colonies, suggesting the increased self-renewal activity of CSCs (Fig. 2C). Although the abilities of HG4 to maintain ZsGreen + CSCs varied depending on the hydrogel’s batches they always maintained CSCs by more than 10-fold, compared to controls HG- and HG5 (Fig. 2D, E) and based on these optimization experiments, PA531-HG4 was identified as a hit hydrogel that mimics the niche for human pancreatic CSCs and PA531-HG5 was used as its control.

### PA531-HG4 binds to serum-derived soluble factors, whose gene expression is correlated with prognosis in PAAD patients

**Fig. 3:**
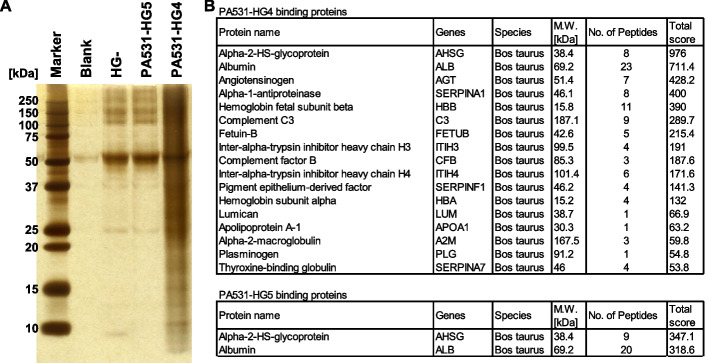
Proteomics analysis of PA531-HG4-binding proteins by mass spectrometry. **A** Representative silver-stained band of HG-binding factors gel after one-dimensional sodium dodecyl sulfate–polyacrylamide gel electrophoresis (SDS-PAGE). **B** PA531-HG4 and -HG5-binding proteins revealed by mass spectrometry. The proteins identified by LC–MS/MS are listed in the order of the sum of their Mascot software scores for each peptide fragment. Detailed information of peptide fragments was shown in Additional file 3 (for PA531-HG4-binding factors) and Additional file 4 (for PA531-HG5-binding factors)

To investigate the mechanisms behind how PA531-HG4 functions as the pancreatic CSC niche, PA531-HG4 binding proteins were analyzed. After KLM1 cells were cultured on the hydrogel-coated coverslips and completely removed by trypsinization, the protein fragments (the so-called hard corona) remaining on the gel surface were analyzed by SDS-PAGE and silver staining. Many proteins were found to bind onto HG4 (Fig. 3A and Figure S3A) with LC–MS/MS analysis revealing that HG- and HG5 bound alpha-2-HS-glycoprotein and albumin, both proteins abundantly present in serum, whereas several types of serum-derived factors, reported to have cancer-promoting functions, were identified on the hit gel PA531-HG4-binding proteins (Fig. 3B). For example, thyroxine-binding globulin (SERPINA7) which binds thyroid hormones in the blood, and thyroid hormones triiodothyronine (T_3_) and thyroxine (T_4_) which have a promoting and inhibitory action on the growth and invasion of pancreatic cancer cells [32–34]. Plasminogen (PLG) was found, which is activated by the plasminogen activator and the broad-spectrum serine protease plasmin, and ultimately leads to the proteolytic degradation of the extracellular matrix (ECM), which is a key biological process that drives tumor cell motility and progression towards the invasive phenotype [35–37]. Alpha-2-macroglobulin (A2M) was also observed, which is a known protease inhibitor [38] but it also functions as a non-specific carrier for various cytokines and growth factors, perhaps implicating its contributions to cancer as a part of the niche [39]. The complement system (C3) plays a key role in cancer progression aside from boosting immunity [40].

**Fig. 4:**
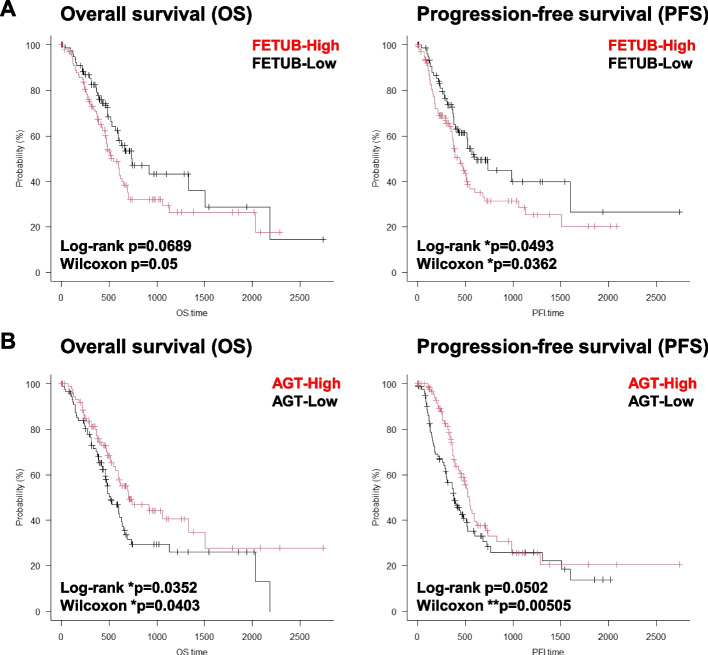
Survival analysis in pancreatic cancer patients categorized by FETUB and AGT gene expression. **A** Kaplan–Meier survival curves of overall survival (OS) and progression-free survival (PFS) comparing two patient groups categorized by FETUB gene expression. The *x* axis represents time (days), while the *y* axis represents the estimated survival probability. The red line represents the cohort of PAAD patients with high FETUB gene expression (*n* = 79), while the black line represents patients with low expression of FETUB (*n* = 102). Log-rank and Wilcoxon test *p* value is provided. *p < 0.05. **B** Kaplan–Meier survival curves of overall survival (OS) and progression-free survival (PFS) comparing two patient groups categorized by AGT gene expression. The red line represents the cohort of PAAD patients with high FETUB gene expression (*n* = 90), while the black line represents patients with low expression of FETUB (*n* = 91). Log-rank and Wilcoxon test *p* value comparing survival between groups are provided. *p < 0.05. **p < 0.01

To narrow down the identified candidate molecules into key targets with clinical significance, the survival data of PAAD patients were downloaded from The Cancer Genome Atlas (TCGA) database and analyzed (Figure S3B). In particular, the high expression of *FETUB* gene in tumors was significantly correlated with worse progression-free survival (PFS) rather than overall survival (OS), suggesting fetuin-B is implicated in PAAD progression and therapeutic resistance (Fig. 4A). For this statistical analysis, PAAD patients were not categorized by location or stage of tumor development. When the correlation between the expression of *FETUB* gene and the prognosis was examined in consideration of patient data such as stage and location, the statistical significance was pronounced particularly in the PAAD located in the tail of the pancreas. In terms of stages of malignancy, the *p*-value is relatively low in stages III and IV, but further analysis is needed due to the small sample size. Gene set enrichment analysis (GSEA) using transcriptome data further demonstrated that patients with high *FETUB* expression enriched a set of genes associated with “xenobiotic metabolism”, suggesting that fetuin-B contributes to the resistance to chemotherapy due to their high detoxification consistent with the PFS data (Figure S3C). On the other hand, angiotensinogen (AGT), a precursor of angiotensin II, was a factor correlated with a better prognosis (Fig. 4B). The *p*-value is low in PAAD located in the head of the pancreas. Thus, AGT expression may be a potential predictor of better prognosis in patients at the N1 stage than at the N0 stage (Figure S4). Angiotensin II is a key molecule associated with the renin-angiotensin system which promotes cancer by inducing angiogenesis, proliferation, and cancer-associated inflammation [41, 42]. Since *AGT* gene expression correlates with good patient prognosis, it seemingly does not logically fit with it being a niche mimicry-binding protein. However, as angiotensinogen is produced in the liver and then secreted into the bloodstream where it plays crucial roles in the renin-angiotensin system [41, 43], we speculate that PA531-HG4 may have an inhibitory effect on this system by trapping angiotensinogen, the precursor of angiotensin II. Consistently, gene expressions of proteins detected in both HG4 and HG5 were not associated with the patients’ outcome (Figure S3D). These results indicate that an approach using niche mimicry could be a promising tool to identify target molecules present in the tumor microenvironment with clinical significance.

## Discussion

In the long-term history of cancer research, we have gained a deep knowledge of cancer biology at the molecular and cellular levels by virtue of various analytical methods. Nevertheless, cancers are still the major leading cause of death globally and part of the explanation is due to the lack of research tools that achieve a comprehensive understanding of the cancer microenvironment. In this study, we successfully fabricated a polymeric hydrogel PA531-HG4 as a niche-mimicking material for pancreatic CSCs and identified new therapeutic targets whose expression has a significant correlation with patients’ survival.

Fetuin-B is a soluble factor derived from the liver. This study has found a high correlation between Fetuin-B gene expression and patient PFS, suggesting potential implications in drug resistance, although the detailed mechanisms remain unclear. Studies have shown that Type 2 diabetes and liver steatosis are associated with elevated levels of fetuin-B in both liver and circulating blood [44, 45]. Although those diseases are associated with pancreatic cancer [46–48], the roles of fetuin-B in cancer are not conclusive. Zhan et al. demonstrated a decrease in proliferation and migration in prostate cancer cells overexpressing *FETUB* [49]. Holm et al. showed that plasma fetuin-B level is increased in colorectal cancer patients and that it correlates with a favorable prognosis [50]. In the model of acute myocardial infarction, fetuin-B has been found to mobilize monocytes and macrophages, and the possibility of its influence on tumor-associated macrophages remains to be investigated [51]. Elucidating these issues could clarify the value of fetuin-B as a novel biomarker and/or therapeutic target.

Polymers have been frequently applied in the field of regenerative medicine in particular for stem cell maintenance and expansion [52, 53]. Considering the mechanisms that polymers act on the regulation of cell behaviors, high affinity with proteins is an important feature of polymers. There is no biological ligand in the polymer microarray system used in this study, but niche-mimicking gel PA531-HG4 highly adsorbs serum proteins from culture media, which bind to cell surface receptors and affect cellular activities [54–56]. Among 17 factors we identified in this study as bound to PA531-HG4, 15 are all closely associated with cancer cell regulation [57–63]. In addition, studies have reported increased concentrations of C3, AHSG, and SERPINF1 proteins in the peripheral blood of pancreatic cancer patients [64, 65]. As they performed, comprehensive proteome analysis using patient blood samples is useful in the search for cancer patient-specific prognostic markers [65–68]. However, the enormous complexity of plasma, serum, and the wide range of protein concentrations make it extremely difficult to quantify its global profile. Considering the fact that CSC niche-mimicking hydrogels trap the same proteins identified by some conventional methods, our data strongly suggest that biomaterials could identify functional biomarkers.

Accumulating evidence shows that cells respond differently to scaffolds depending on their physical and chemical properties [69]. Pancreatic cancer tissue has also been shown to exhibit unique physical properties [70, 71]. In this study, PEG-crosslinked 3D hydrogels coated on glass coverslips mimic CSC niches more closely than simple polymer coatings with hydrogelation technology conferring new physical properties onto the 2D polymer scaffolds. Additional studies will be required to determine the contribution of the various physicochemical properties of the polymer hydrogel, such as wettability, mechanical stiffness, and surface topography.

## Conclusion

An artificial niche screen (i.e., niche mimicry) for pancreatic CSCs was created by polymer microarray and the biological activities were optimized by hydrogelation. Proteome analysis of binding proteins showed that the niche-mimicking hydrogel traps multiple serum factors, one of which was closely associated with poor outcomes in PAAD patients. This study demonstrated the usefulness of synthetic polymer materials in identifying cancer biomarkers/therapeutic targets.

### Supplementary Information

**Additional file 1: Figure S1.** Experimental design to identify niche-mimicking materials using two polymer microarray slides. **Figure S2.** Comparison of niche-mimicking abilities in five PA531 hydrogels. **Figure S3.** Statistical analysis of pancreatic cancer patients classified by gene expression of indicated factors evaluated by log-rank and Wilcoxon test. **Figure S4.** *p*-values of Kaplan-Meier Survival Analysis by gene expression levels of FETUB or AGT categorized by tumor location and stages.

**Additional file 2.** List of all fluorescent intensity values.

**Additional file 3.** PA531-HG4 binding factors.

**Additional file 4.** PA531-HG5 binding factors.

**Additional file 5.** List of genes highly expressed.

## Data Availability

The datasets are available from the corresponding author upon reasonable request.

## Abbreviations

CSC: Cancer stem cell
DEAEA: 2-(Diethylamino)ethyl acrylate
DEAEMA: 2-(Diethylamino)ethyl methacrylate
DMAAn: N,N-dimethyl acrylamide
FACS: Fluorescence-activated cell sorting
GSEA: Gene set enrichment analysis
GMA: Glycidyl methacrylate
HG: Hydrogel
LC–MS/MS: Liquid chromatograph-mass spectrometry
MAn: N,N-methylaniline
MEMA: 2-Methoxyethyl methacrylate
MMA: Methyl methacrylate
NMP: N-Methyl-2-pyrrolidon
PA: Polyacrylate
PAAD: Pancreatic adenocarcinoma
PEG: Polyethylene glycol
PEGDA: Poly(ethylene glycol)diacrylate
PU: Polyurethane
THFFMA: Tetrahydrofurfuryl methacrylate
TMSPMA: 3-(Trimethoxysilyl)propyl methacrylate
