# Supplementary material for: A niche-mimicking polymer hydrogel-based approach to identify molecular targets for tackling human pancreatic cancer stem cells
Source: Inflamm Regen. 2023 Sep 27;43:46. doi: 10.1186/s41232-023-00296-0 (PMC10523636; doi:10.1186/s41232-023-00296-0)
Supplement: Supplementary file 1 — Additional file 1: Figure S1. Experimental design to identify niche-mimicking materials using two polymer microarray slides. Figure S2. Comparison of niche-mimicking abilities in five PA531 hydrogels. Figure S3. Statistical analysis of pancreatic cancer patients classified by gene expression of indicated factors evaluated by log-rank and Wilcoxon test. Figure S4. p-values of Kaplan-Meier Survival Analysis by gene expression levels of FETUB or AGT categorized by tumor location and stages. [file 41232_2023_296_MOESM1_ESM.pdf]

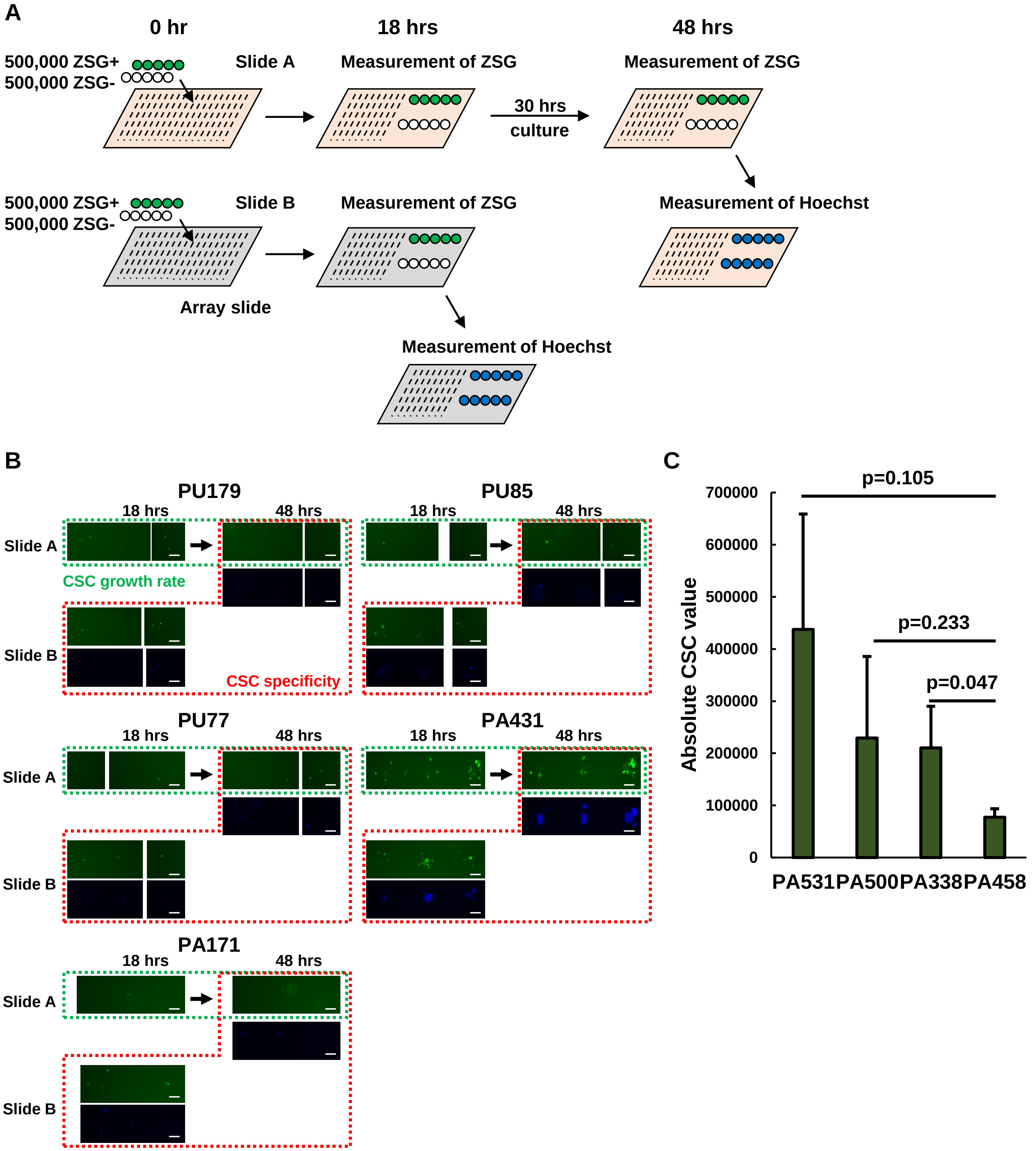
A
0 hr
18 hrs
48 hrs
500,000 ZSG+
500,000 ZSG-
Slide A
Measurement of ZSG
30 hrs
culture
Measurement of ZSG
Measurement of Hoechst
500,000 ZSG+
500,000 ZSG-
Slide B
Measurement of ZSG
Array slide
Measurement of Hoechst
B
PU179
18 hrs
48 hrs
Slide A
CSC growth rate
Slide B
CSC specificity
PU85
18 hrs
48 hrs
PU77
18 hrs
48 hrs
Slide A
Slide B
PA431
18 hrs
48 hrs
PA171
18 hrs
48 hrs
Slide A
Slide B
C
p=0.105
p=0.233
p=0.047
Absolute CSC value
700000
600000
500000
400000
300000
200000
100000
0
PA531
PA500
PA338
PA458

Figure S1

**Figure S1. Experimental design to identify niche-mimicking materials using two polymer microarray slides.**

**A**: Schematic representation of the experimental design of niche-mimicking polymer screening. Five hundred thousand ZsGreen+ KLM1 CSCs and ZsGreen- KLM1 non-CSCs were seeded onto two slides with the same set of polymers (Slide A and B). After incubation for 18 hrs, the fluorescence intensities of ZsGreen in both slides were measured, and only Slide A was kept incubating for up to 48 hr. In contrast, Slide B was fixed at 18 hr, stained with Hoechst 33258, and then the fluorescence intensities of Hoechst 33258 were measured. At 48 hr, the fluorescence intensities of ZsGreen and Hoechst 33258 in Slide B were measured. ZSG: ZsGreen.

**B**: Fluorescence images of cells at 18 and 48 hrs after culturing on polymers with seemingly high value of "CSC growth rate" but excluded as hit. Scale bar=200 μm.

**C**: Comparison of fluorescence intensities of ZsGreen at 48 hrs. Data represent the averages±standard error (SE) from three polymer spots.

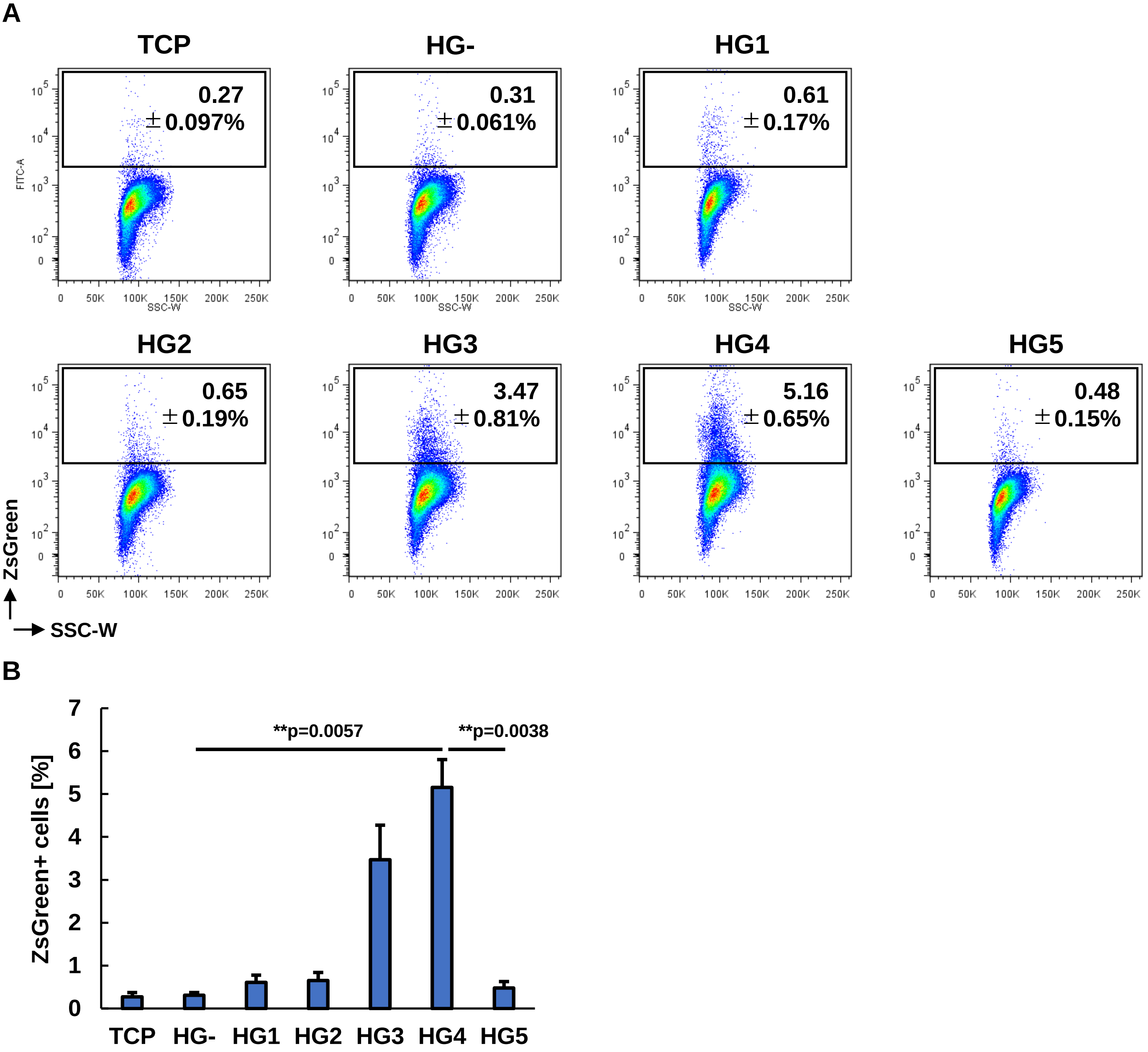


**Figure S2. Comparison of niche-mimicking abilities in five PA531 hydrogels.**

**A**: FACS plots of KLM1-Gdeg cells related to Figure 2B. KLM1-Gdeg cells were cultured on the indicated hydrogels for 5 days. TCP: Tissue culture polystyrene.

**B**: The mean percentages±SD of ZsGreen+ cells are shown in plots and displayed in a bar graph. **P<0.01.

**Figure S2**

**A**

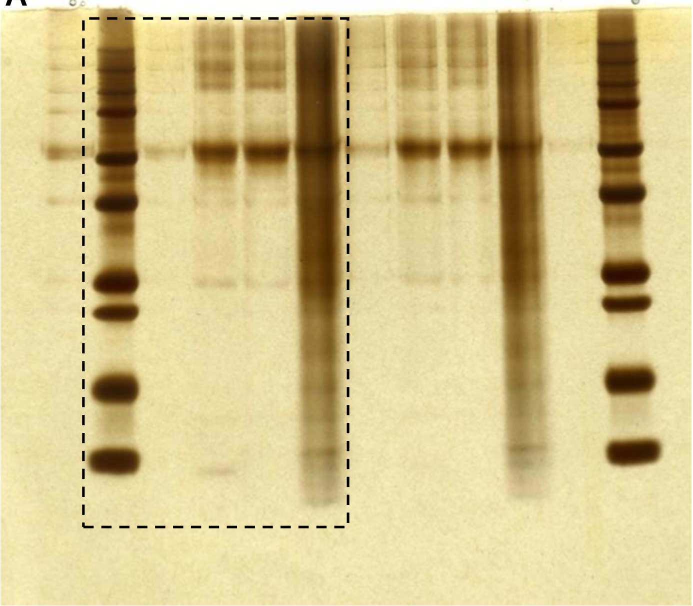

**B**

| Gene symbol | OS Log-rank | OS Wilcoxon | PFS Log-rank | PFS Wilcoxon |
|---|---|---|---|---|
| FETUB | 0.0689 | 0.05 | 0.0493 | 0.0362 |
| AGT | 0.0352 | 0.0403 | 0.0502 | 0.00505 |
| SERPINA1 | 0.848 | 0.853 | 0.367 | 0.503 |
| HBB | 0.248 | 0.22 | 0.34 | 0.411 |
| C3 | 0.611 | 0.611 | 0.129 | 0.277 |
| ITIH3 | 0.725 | 0.226 | 0.677 | 0.544 |
| CFB | 0.106 | 0.065 | 0.45 | 0.486 |
| ITIH4 | 0.963 | 0.916 | 0.885 | 0.755 |
| SERPINF1 | 0.737 | 0.873 | 0.245 | 0.572 |
| HBA | 0.761 | 0.875 | 0.505 | 0.911 |
| LUM | 0.468 | 0.958 | 0.367 | 0.991 |
| APOA1 | 0.324 | 0.519 | 0.781 | 0.846 |
| A2M | 0.569 | 0.274 | 0.604 | 0.142 |
| PLG | 0.158 | 0.157 | 0.155 | 0.116 |
| SERPINA7 | 0.828 | 0.876 | 0.967 | 0.951 |

**C**

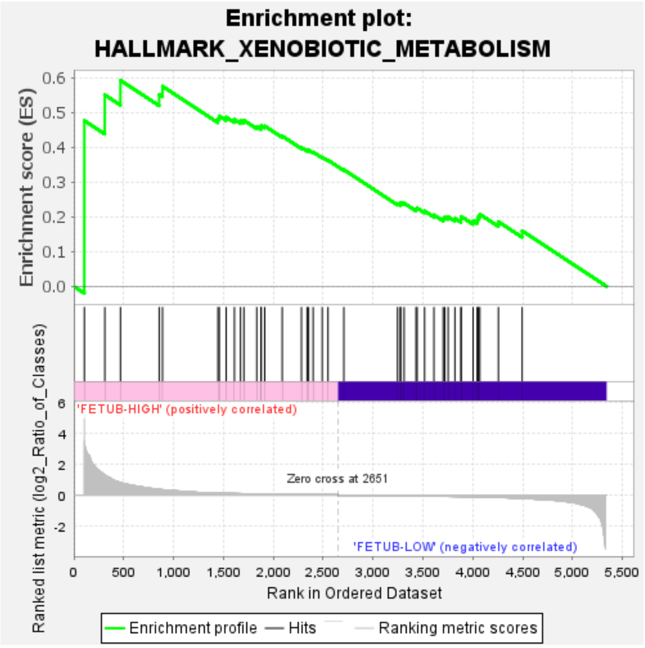


**D**

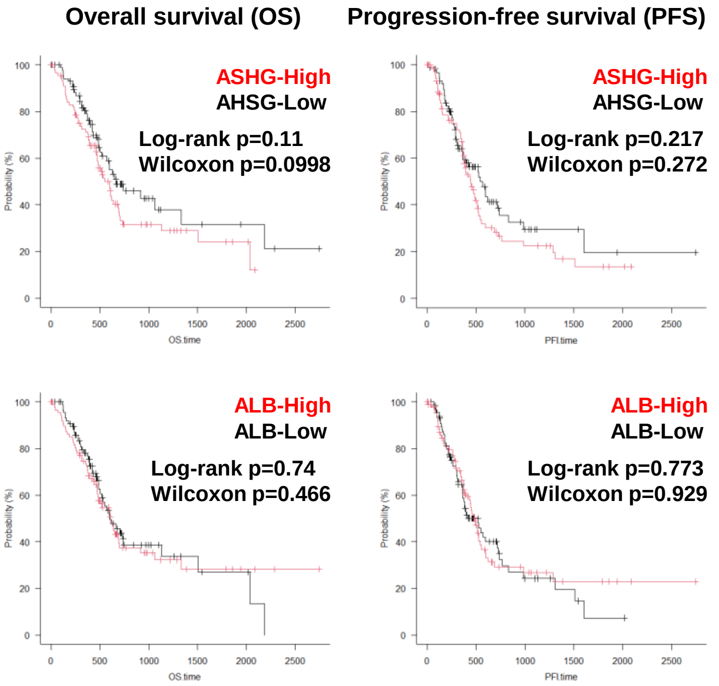


**Figure S3**

**Figure S3. Statistical analysis of pancreatic cancer patients classified by gene expression of indicated factors evaluated by log-rank and Wilcoxon test.**

**A:** An original image of the silver-stained gel used in Figure 3A. The dotted square indicates the cropped area.

**B:** Log-rank and Wilcoxon test for genes of PA531-HG4-binding factors. The p-value was calculated by the comparative statistical analysis of PFS and OS among patients classified according to the gene expression level of each factor.

**C**: Gene set enrichment analysis (GSEA) of differentially expressed genes between $FETUB^{High}$ and $FETUB^{Low}$ PAAD patients. The normalized FKPM (fragments per kilobase of transcript per million mapped reads) gene expression data from TCGA pancreatic cancer (PAAD) cohort was obtained (downloaded 2022 July 6) and categorized into two ($FETUB^{High}$ and $FETUB^{Low}$) based on the expression levels of FETUB gene. Totally 7209 differentially expressed genes with a student's t-test p-value of less than 0.05 between $FETUB^{High}$ and $FETUB^{Low}$ were extracted and performed GSEA analysis to identify enriched gene set associated with each group. In our analysis, we used GESA 4.3.2 software, a publicly available application from the Broad Institute. Gene set file used was h.all.v2023.1.Hs.symbols.gmt and Human_Gene_Symbol_with Remapping_MSigDB.v2023.1.Hs.chip for collection chip file to obtain predefined gene sets based on the gene ontology annotations. The Log2_Ratio_of Classes metric was used for ranking genes. Gene sets with an FDR (false discovery rate) below 25% and nominal p-value below 0.05 were considered statistically significant. The detailed enrichment results were shown in Additional file 5.

**D**: Kaplan-Meier curves of overall survival (OS) and progression-free survival (PFS) for patients with high or low expression of AHSG (top two panels) and ALB (bottom two panels), proteins non-specifically bound with PA531-HG4 and -HG5. The red line represents the cohort of PAAD patients with high gene expression (ASHG n=88, ALB n=90), while the black line represents patients with low expression (ASHG n=93, ALB n=91). Log-rank and Wilcoxon test p-value comparing survival between groups are provided.

| | | FETUB High vs Low | | AGT High vs Low | |
|---|---|---|---|---|---|
| | | OS | PFS | OS | PFS |
| Location | Body | 0.601 | 0.983 | 0.0513 | 0.36 |
| | Head | 0.0512 | 0.483 | 0.0833 | **0.0463** |
| | Tail | **0.0497** | 0.0522 | 0.651 | 0.401 |
| AJCC Stage | Ⅰ | 0.483 | 0.345 | 0.931 | 0.534 |
| | ⅡA | 0.375 | 0.46 | 0.768 | 0.854 |
| | ⅡB | 0.0639 | 0.871 | 0.0687 | **0.0413** |
| | Ⅲ & Ⅳ | 0.083 | **0.0269** | 1 | 0.157 |
| AJCC T | T1 | 0.0701 | 0.864 | 0.919 | 0.608 |
| | T2 | 0.863 | 0.669 | 0.484 | 0.463 |
| | T3 | 0.0753 | 0.855 | 0.144 | 0.422 |
| AJCC N | N0 | 0.592 | 0.476 | 0.686 | 0.795 |
| | N1 | 0.133 | 0.913 | **0.00853** | **0.022** |

Tail of pancreas

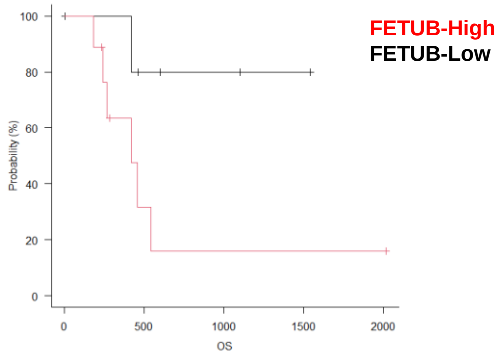


AJCC Stage Ⅲ and Ⅳ

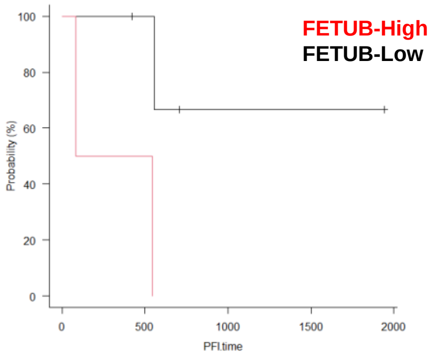


Head of pancreas

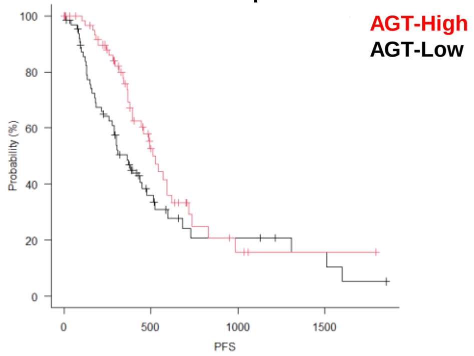


AJCC Stage ⅡB

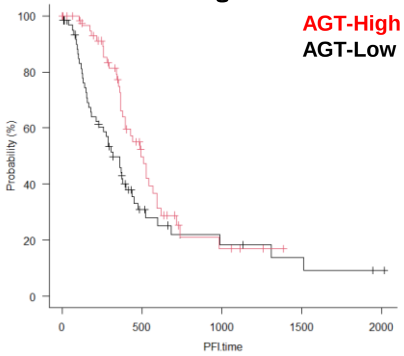


AJCC N

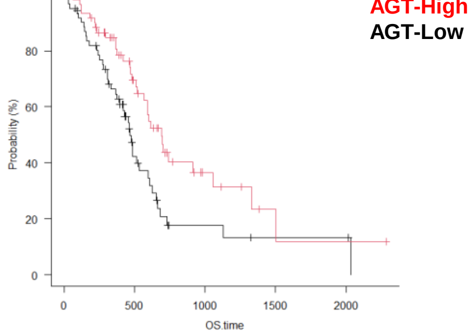


AJCC N

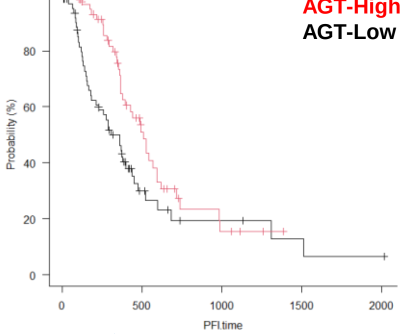


**Figure S4**

**Figure S4. p-values of Kaplan-Meier Survival Analysis by gene expression levels of FETUB or AGT categorized by tumor location and stages.**

Kaplan-Meier survival analysis of two groups categorized by gene expression levels of FETUB or AGT in different tumor locations and stages. Table of the p-value from the log-rank test of patients with high and low gene expression of FETUB and AGT based on The American Joint Committee on Cancer (AJCC) Cancer Staging Manual was shown in the upper panel. p-values less than 0.05 are bolded in Table and their Kaplan-Meier survival curves are shown in lower panels.
